# A Comparison of Accommodative Ability in Healthy Controls, Diabetics, and Healthy Subjects with a Family History of Diabetes

**DOI:** 10.22599/bioj.438

**Published:** 2025-09-22

**Authors:** Suchismita Rout, Aiswaryah Radhakrishnan

**Affiliations:** 1 Faculty of Medicine and Health Sciences, SRM Institute of Science and Technology, Kattankulathur, India

**Keywords:** Diabetes mellitus, accommodation, diabetes, family history of diabetes, prediabetes

## Abstract

**Background:**

The aim of this study was to compare accommodative amplitude (AA) and accommodative facility (AF) in healthy subjects with a family history of diabetes (FHD+), individuals with diabetes mellitus (DM), and healthy controls (HC).

**Material and methods:**

This cross-sectional, observational, comparative study was conducted among 89 subjects who attended in the age group between 30 and 40 years. The subjects were categorised into three groups: 30 healthy controls (HC) (mean age: 35.1 ± 4.5 years), 31 healthy subjects with a family history of diabetes (FHD+) (35.5 ± 3.2 years) and 28 subjects diagnosed with diabetes (DM) (36.5 ± 3.5 years). An informed consent form was obtained from subjects before conducting procedures. The amplitude of accommodation was assessed using the minus-lens technique. Additionally, accommodative facility was evaluated monocularly and binocularly using ± 1.50DS flippers. The effects of age, fasting plasma glucose levels, and glycated haemoglobin levels on accommodative parameters were examined using multiple regression analysis. One-way ANOVA with the Bonferroni post hoc test was used to test for significant differences in accommodative parameters.

**Results:**

The mean amplitudes of accommodation for the three groups were statistically significant (Mean AA_DM_: 3.4 ± 1.0; Mean AA_FHD+_: 4.63 ± 0.83; Mean AA_HC_: 6.25 ± 1.33; p = 0.001). Similarly, the mean monocular accommodative facility (AF) for the three groups differed significantly (mean AF_DM:4.35_ ± 1.34, mean AF_FHD+_: 5.95 ± 1.4; mean AF_HC:7.65_ ± 1.18cpm; p = 0.001). In multiple regression, age nearly significantly affected AF in the FHD+ group, with (R^2^ = 0.492, p = 0.040). Whereas, age and FBS were the predictors of AA in FHD+ (R^2^ = 0.598, p = 0.001; R^2^ = 0.400, p = 0.026).

**Conclusions:**

Healthy subjects with a family history of diabetes who are at increased risk of developing prediabetes had reduced accommodations. The AA and AF values are notably lower than the expected value for this age group. Identifying and monitoring these individuals could provide an opportunity for early intervention, potentially delaying the progression of accommodative anomaly-like symptoms associated with DM. This observation highlights the importance of considering family history and prediabetic status when examining accommodative function.

## Introduction

Diabetes mellitus (DM) is a group of metabolic diseases characterised by chronic hyperglycaemia. Stemming from inadequate insulin production or action, this condition disrupts the body’s processing of carbohydrates, proteins and fats (Association, 2006). Erstwhile, it is predicted that the global prevalence of DM was 9.3% (463 million) in 2019 and is expected to increase to 10.9% (700 million) in 2045 (Saeedi et al., 2019), with DM increasingly affecting young individuals, characterised by insulin resistance and impaired insulin secretion, presenting a concerning trend (Saeedi et al., 2019). Several factors are responsible for the increased incidence of diabetes. Key predisposing factors include obesity, a familial history of diabetes, and a sedentary lifestyle, which collectively play a significant role in heightening the risk of diabetes development (Lascar et al., 2018). Diabetes can have a significant impact on the eye, especially the posterior and anterior regions. It can cause diabetic retinopathy, neuropathy, glaucoma, and macular oedema (Nentwich and Ulbig, 2015; Sayin et al., 2015). In anterior segments, complications can include corneal issues. These include abnormal sensitivity, delayed healing, diabetic keratopathy, and nerve damage. They may also cause early-onset cataracts (Han et al., 2019).

Impacts of DM also manifest at a visual functional level (Ismail and Whitaker, 1998). An earlier study reported that a rise in glucose can alter the refractive index of the ocular media. As a result, such modifications may increase refractive power and cause transient changes in vision (Khan et al., 2017). Existing research suggests that diabetes itself alters the lens’s structure, regardless of changes in glucose levels (Abokyi et al., 2021; Calvo-Maroto et al., 2014). According to previous investigations, some diseases, including multiple sclerosis (MS) and DM, may cause the deterioration of accommodative functions at earlier ages (Küçük et al., 2019; Sırakaya et al., 2020). Among them, DM, a common metabolic disorder, leads to severe ocular consequences, including impaired accommodative function, resulting from multiple underlying pathological processes (Sırakaya et al., 2020). With regard to accommodative functions, authors suggested (Abokyi et al., 2021; Abokyi et al., 2016) diabetes can interfere with focussing ability, subsequently leading to reduced accommodative amplitude. This difficulty of focussing on near objects arises due to a loss of elasticity in the lens (Silva-Viguera and Bautista-Llamas, 2023). Previous researchers have proposed that individuals with diabetes have reduced ability to adjust their focus compared to those without diabetes, and this may be linked to glycaemic control (Abokyi et al., 2021). Furthermore, diabetes is associated with reduced accommodative functions, as demonstrated in another investigation by Sırakaya *et al*. (2020), which compared the accommodative functions of young diabetics and healthy subjects using the minus lens technique to measure amplitude of accommodation. They found that diabetics had 1D less accomodation than non-diabetics. The reduction in accommodative functions is dependent on the glycosylated haemoglobin level (Abokyi et al., 2021; Abokyi et al., 2016). However, it is uncertain whether similar accommodative changes occur in individuals with a family history of diabetes. A family history of diabetes is a well-established risk factor for its development (Hemminki et al., 2010). Individuals with a first-degree relative (parent or sibling) with diabetes are particularly at increased risk of developing the condition themselves. Consequently, the number of individuals with prediabetes, known as a precursor of DM, is increasing annually. Existing research highlights that the presence of visual functional alterations, such as impaired colour discrimination and contrast sensitivity, can prevail at the prediabetic stage (Chande et al., 2020; Karson et al., 2020). However, these studies (Chande et al., 2020; Karson et al., 2020) have not accounted for the potential impact of family history of diabetes. The substantial risk associated with a positive family history of DM can lead to the progression from prediabetes to overt diabetes. However, studies are scarce to assess accommodative functions in healthy subjects with a family history of diabetes. Taking this perspective into account, the current study speculates that since healthy subjects with a family history of diabetes (FHD+) who are prediabetic may experience similar visual alterations, such as accommodative anomaly, which has not been addressed in previous literature.

To address this gap, this present study aimed to compare accommodative functions between healthy subjects with a family history of diabetes (FHD+) in comparison to those with diagnosed DM and healthy controls (HC).

## Materials and Methods

### Study populations and design

A cross-sectional, observational and comparative study was carried out in the optometry facilities of SRM Medical College Hospital and Research Centre, India, between November 2021 and December 2022. This study adhered to the principles of the Declaration of Helsinki and received approval from the Institutional Ethics Committee (IEC/2020/2168). The study selected subjects with DM, FHD+, and HC in an age group between 30 and 40 years. This age range was chosen based on the previous research (Anjana et al., 2015), which reported the mean age of developing diabetes somewhere between the ages of 30 and 40 years. According to earlier research by Chattopadhyay and Seal (1984), the mean age of onset for presbyopia is reported to be between 30 and 40 years. Therefore, we aimed to assess the accommodative functions of individuals within this specific age range, focussing on those with FHD+ who are at risk of developing prediabetes, and comparing them to individuals with DM and HC. After obtaining the informed consent forms, all the subjects underwent detailed ophthalmic evaluation, including a dilated fundus examination, to rule out other retinopathy abnormalities. The fasting plasma glucose (FPG) and HbA1c values were collected from the subjects’ medical records, which were obtained from the outpatient department of SRM Hospital. These values were recorded prior to the study procedure and were used to determine the subjects’ glycaemic status. Specifically, the FPG values were obtained from the subjects’ laboratory reports, which were generated as part of their routine medical care. The HbA1c values were also obtained from the subjects’ laboratory reports and were used to assess their average blood glucose levels over the preceding 2–3 months. The medical records were accessed with the subjects’ consent.

### Inclusion criteria

The subjects were classified based on their FPG according to American Diabetes Classifications (ADA).

**HC**: FPG ≤100 mg/dL, and either with no evidence of diabetes or no family history of diabetes.

**DM:** FPG≥125 mg/dL and diagnosed as diabetes by a diabetologist or self-reported medical record.

**FHD+**: Subjects with FHD+ required three or more recognised factors for prediabetes (age ≥35 years, a FPG level of 100–125 mg/dL, and an HbA1c of 5.7–6.4%, as well as a family history of diabetes with first-degree relatives with either or both parents), as per the ADA guidelines (American Diabetes Association, 2022). The study included subjects who had distance visual acuity better than or equal to 0.2 logMAR (6/9 on the Snellen chart) in all three groups.

### Exclusion criteria

Subjects with a history of significant ocular disease, cataracts, glaucoma, myopia of more than 6D, a history of intraocular surgery or medication, PDR with diabetic macular oedema, a history of using any systemic medications that can affect accommodation, defective pupillary reflexes, and subjects who were uncooperative were excluded.

### Accommodative examination procedure

To evaluate the accommodative function in our study group, we employed two standardised tests: Amplitude of Accommodation (AA) and Accommodative Facility (AF). These tests were chosen because they provide a comprehensive assessment of the accommodative system, allowing us to evaluate both the maximum accommodative response (AA) and the ability to rapidly change focus between near and far objects (AF). The AF test can evaluate the stamina and dynamics of accommodative response (Scheiman and Wick, 2015). The AA procedure was conducted under adequate lighting conditions at 40 cm using an N8 target that can be easily read by the subjects.

### AA

AA was measured monocularly using the minus lens method. Minus lenses were added in the trial frame in –0.25D steps until the subject reported sustained blur that could not be cleared by additional conscious effort. Subjects were allowed up to 5–10 s for each lens presentation to clear the letters. The amplitude of accommodation was firstly measured in the right eye, followed by left eye. The total AA was documented as the total dioptric equivalent of working distance (i.e., 2.50 D) plus the amount of minus lens power added. The minus lens method was chosen over the push-up method in this study due to its distinct advantages. This method has demonstrated high repeatability in previous studies (Chen et al., 2019; Silva-Viguera and Bautista-Llamas, 2023). Despite its reduction in image size, it enables subjects to detect focus earlier, leading to lower but more accurate AA measurements. Moreover, multiple studies have confirmed the minus lens method’s superior repeatability, minimising intra-measurement errors (Antona et al., 2009; García-Montero et al., 2019; Momeni-Moghaddam et al., 2014).

### Accommodative facility

Accommodative facility was measured monocularly and then binocularly using hand-held flippers (±1.50 DS). Using +1.50/-1.50DS flippers allowed us to assess accommodative function across the entire age range of 30–40 years while minimising potential fatigue and discomfort. Measurements were taken in the right eye first, while the left eye remained occluded. The test was carried out for one minute, and the total number of cycles (each occurrence of clearing both sides of the lenses was considered as one cycle) was recorded as cpm. During the procedure, subjects were instructed to hold the near vision card at 40 cm, clear the letters as quickly as possible with each flip, and verbally confirm when the letters were clear by saying ‘clear’. The accommodative target of N8 was viewed at 40 cm by each eye independently and could be read by subjects effortlessly. This test could help to identify one of the following clinical accommodative anomalies, such as accommodative insufficiency or accommodative infacility, as reported by available researchers (Balke et al., 2022; Lara et al., 2001).

### Statistical analysis

Statistical analyses were performed with SPSS Version 20.0 Chicago, IL, USA. The normality of data distribution was tested using the Shapiro-Wilk test. Results were presented with mean and standard deviation in each group. The difference in AA and AF between the three groups was analysed using a one-way ANOVA. The independent factors were the groups (HC, FHD+, and DM), while the dependent factors were the accommodative amplitude and accommodative facility. Post-hoc analyses were performed using the Bonferroni correction to determine significant differences between groups. Multiple regression analysis was performed to identify predictors of Accommodative functions p values less than 0.05 were considered to indicate statistical significance.

## Results

**Figure 1:**
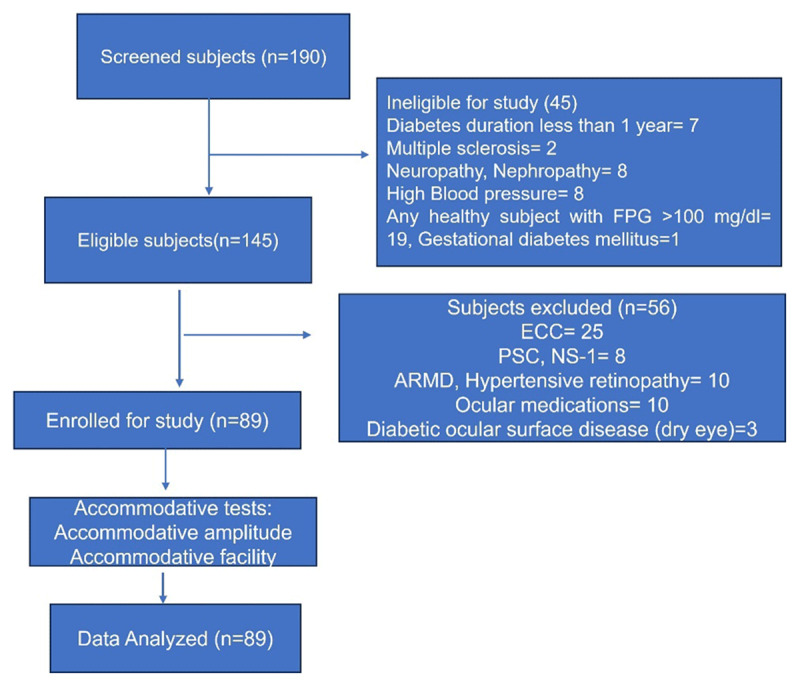
Illustrates the flow chart for selection of study subjects (Abbreviations: ECC: Early cataract changes, FPG: Fasting plasma glucose, ARMD: Age-related macular degeneration, PSC: posterior subcapsular cataract, NS: Nuclear sclerosis-grade 1).

The subject selection process is illustrated in Figure 1. Following a rigorous screening and selection process, the concluding sample of 89 subjects, aged 30–40 years, were included in the final analysis. The sample comprised 31 (34.34%) subjects with HC, 30 (33.7%) subjects with FHD+, and 28 (31.4%) subjects with DM. The demographic and clinical characteristics of the study are summarised in Table 1. The distribution of age was identical between the groups (p = 0.401). Among the diabetics, the mean duration of diabetes was 5.3 ± 2.4 years. The ANOVA test revealed a significant difference in FBS and HbA1c between the groups (p = 0.001).

**Table 1:** Comparison of subject’s demographics and clinical characteristics.


VARIABLES	HC (n = 31)	FHD+ (n = 30)	DM (n = 28)	p VALUES

Age, years	35.1 ± 4.5	35.5 ± 3.2	36.5 ± 3.5	0.401

FBS, mg/dl	92.5 ± 4.6	112 ± 7.7	163.2 ± 23.13	**0.001**ǂ

HbA1c (%)	5.2 ± 0.19	5.4 ± 0.11	8.2 ± 2.17	**0.001**ǂ

Disease duration (years)	–	–	5.3 ± 2.4	–

Distance VA	0.03 ± 0.07	0.01 ± 0.05	0.12 ± 0.13	**0.001**ǂ


Abbreviations: HC, healthy control; FHD+, family history of diabetes; DM, diabetes mellitus; FPG, fasting plasma glucose; HbA1c: haemoglobin A1c; VA, visual acuity. Value is presented as mean ±SD, ǂ ANOVA test, Bold values indicate the p value <0.05.

### Amplitude of accommodation

**Figure 2:**
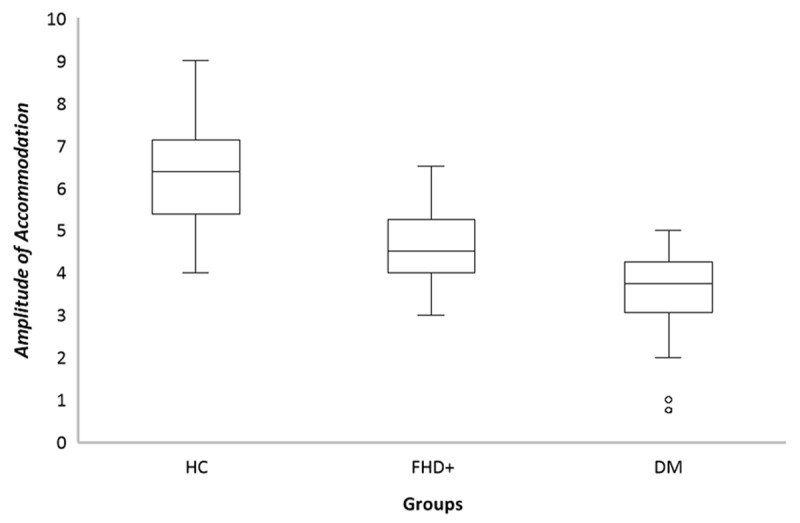
Comparison of amplitude of accommodation between three groups. Box plots of distributions of AA measurements based on minimum, first quartile, second quartile, third quartile, and maxima per sample. The horizontal bold line inside the boxes shows the median, the whisker above and below shows the maxima and minima. The IQR range spans the first and third quartiles.

Figure 2 illustrates comparisons of monocular (right eye) AA differences between the subjects. There was no significant difference between the right eye (RE) and the left eye (LE) (p = 0.916); therefore, only the RE data has been analysed further. The one-way ANOVA test reveals a statistically significant difference (F (2,86) = 48.93, p = 0.001) in the AA among the three groups: healthy controls (HC: 6.25 ± 1.33D), those with FHD+ (4.63 ± 0.83D), and DM (3.4 ± 1.0D). The post-hoc comparisons reveal that all groups differ significantly from each other. Specifically, the HC and FHD+ groups differ significantly from each other (p = 0.001), whereas the FHD+ and DM groups differ significantly (p = 0.002), and the DM and HC groups also differ significantly (p = 0.001), demonstrating a progressive decline in AA from the HC group to the FHD+ and DM groups. These results indicate that the DM group has the lowest AA, followed by the FHD+ group, while the HC group displays the highest AA.

### Accommodative facility

**Figure 3:**
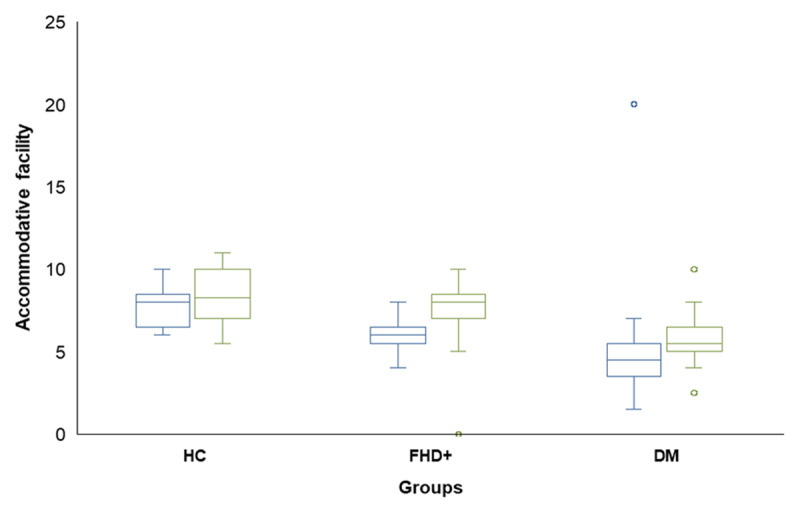
Comparisons of AF measured in cycle/minute between three groups in RE and BE. Box plots of distributions of AF measurements based on minimum, first quartile, second quartile, third quartile, and maxima per sample. The horizontal bold line inside the boxes shows the median, the whisker above and below shows the maxima and minima. The IQR range spans the first and third quartiles.RE, right eyes; BE: Both eyes). Blue box indicates RE and green box indicates BE.

Figure 3 presents a box-and-whisker plot comparison of AF across three groups: HC, FHD+, and DM. There was no significant difference between RE and LE (p = 0.216); therefore, only the RE data has been analysed further. The mean monocular accommodative facility in RE for the HC was 7.65 ± 1.18 cpm, the FHD+ was 5.95 ± 1.4 cpm, and the DM was 4.35 ± 1.34 cpm. One-way ANOVA results indicate significant differences in accommodative facility between the three groups in the RE (F (2,86) = 59.164, p < 0.001) and both eyes (BE) (F (2,86) = 20.312, p < 0.001). Post-hoc Bonferroni analysis reveals significant differences between the HC and FHD+ groups (p = 0.001), the FHD+ and DM groups (p = 0.002), and the DM and HC groups (p = 0.001). These results indicate a progressive decline in accommodative facility from the HC group to the FHD+ and DM groups.

Table 2 represents the results of a multiple regression analysis examining variables associated with accommodative amplitude and accommodative facility in individuals with a family history of diabetes (FHD+) and DM. The β values indicate the estimated change in FHD+ risk associated with a one-unit change in the predictor variables. Age had a β of –0.781, indicated that a higher age was associated with lower accommodative amplitude. FBS had a β of –0.193, also indicating an inverse relationship with AA. Both age (p = 0.001) and FBS (p = 0.026) are important factors related to reduced accommodative amplitude in FHD+ subjects.

**Table 2:** Multiple regression model to predict accommodative amplitude and accommodative facility in FHD+ and DM.


	PREDICTORS	β	R^2^ VALUE	P VALUE	95% CI VALUE

**Variables for FHD**					

AA	Age	–0.781	0.598	0.001	[–0.139, –0.090]

	FBS	–0.193	0.400	0.026	[–1.850, –0.123]

AF	Age	–0.197	0.492	0.040	[–0.228, –0.166]

	FBS	–0.060	0.272	0.065	[–2.225, 0.105]

**Variables for DM**					

AA	Age	–0.263	0.439	0.018	[–0.066, –0.006]

	HbA1c	–0.147	0.191	0.177	[–0.192, 0.036]

	FBS	0.078	0.079	0.472	[–0.006, 0.014]

	Duration	–0.270	0.282	0.015	[–0.153, –0.017]

AF	Age	–0.179	0.079	0.128	[–0.076, 0.010]

	HbA1c	0.043	0.066	0.706	[–0.132, 0.194]

	FBS	–0.234	0.285	0.045	[–0.029, 0.000]

	Duration	0.013	0.185	0.110	[–0.076, 0.010]


For DM, age (β = –0.263, p = 0.018) and duration (β = –0.270, p = 0.015) were significant negative predictors for accommodative amplitude. In contrast, FBS (β = –0.234, p = 0.045) was the only factor identified as influencing accommodative facility. The results suggest that older age and longer diabetes duration are associated with lower accommodative amplitude in individuals with diabetes. These findings imply that age, disease duration, and FBS are important factors related to reduced accommodative ability in diabetic populations.

## Discussion

The current study revealed a significant progressive decline in accommodative amplitude from HC (6.25 ± 1.33D) to FHD+ (4.63 ± 0.83D) and DM groups (3.4 ± 1.0D), with all comparisons showing statistical significance (p = 0.001). Similar patterns were observed in an accommodative facility across monocular and binocular conditions in both FHD+ and DM. The analysis identified distinct predictive factors for accommodative function in different groups. In the FHD+ group, age and FBS levels emerged as key predictors, while the DM group showed significant associations with age, disease duration, and fasting plasma glucose levels. The FHD+ group demonstrated significant differences in accommodative parameters compared to healthy controls, independent of age. These findings emphasise the importance of metabolic control in preserving accommodative function and imply that comprehensive accommodative assessment should be an integral part of prediabetic ocular assessments.

While several studies have investigated accommodative changes in diabetic patients (Abokyi et al., 2016; Silva-Viguera and Bautista-Llamas, 2023; Sırakaya et al., 2020), our research breaks new ground by identifying accommodative anomalies in FHD+ subjects before the onset of diabetes. This is particularly significant, as these subjects were specifically categorised based on their FBS levels at the time of testing, an approach not previously undertaken in other studies.

These findings have important clinical implications. The presence of accommodative anomalies in the FHD+ group suggests their potential as an early screening tool for pre-diabetic changes. The progressive nature of the decline emphasises the importance of regular monitoring for both FHD+ and diabetic subjects. The significant impact of metabolic factors highlights the importance of glycaemic control in preserving accommodative function. Therefore, comprehensive accommodative assessment should be considered an integral part of diabetic eye examinations, potentially serving as a marker for diabetes-related visual dysfunction.

The current study revealed several interesting findings. A progressive decline in accommodative functions was observed from HC to FHD+ and DM patients. Accommodative dysfunction was present in the FHD+ group, suggesting its potential as an early screening tool for pre-diabetic changes.

The AA in the FHD+ and DM groups is approximately 2.25D less than HC; this difference can be considered a clinically significant difference. When measuring AA, various methodological considerations must be taken into account. While both push-up and minus lens methods are commonly employed, research (Antona et al., 2009; Chen et al., 2019) has reported that the push-up method yields higher AA values compared to the minus lens technique.

Several researchers, including Chen *et al*. (2019) and Momeni-Moghaddam *et al*. (2014), have noted that the push-up method tends to overestimate the true value of AA. This overestimation can be attributed to primary factors: the eye’s depth-of-focus and measurement errors associated with close working distances, as highlighted by Antona *et al*. (2009) and Ostrin and Glasser (2004). In contrast, the minus lens method offers distinct advantages. While it does reduce the image size, which enables the subject’s ability to detect focus earlier, resulting in a lower but more accurate AA measurement. Furthermore, multiple studies (Antonaf et al., 2009; García-Montero et al., 2019; Momeni-Moghaddam et al., 2014) have documented that the minus lens method demonstrates superior repeatability, thereby minimising intra-measurement errors.

The linear regression models revealed that FBS was the second significant parameter influencing AA in FHD+, after age. Furthermore, a linear regression analysis demonstrated that AA was significantly associated with age and duration of diabetes in DM subjects, corroborating with results of previous studies (Silva-Viguera and Bautista-Llamas, 2023).

Meanwhile, previous investigations (Adnan et al., 2014; Abokyi et al., 2016) observed significant associations between AA and glycaemic control indicators, such as fasting glucose and HbA1c. The relatively short duration of diabetes in our study population may have limited our ability to detect an association between AA and hyperglycaemia. In contrast, another investigation by Nabovati *et al*. (2022) found a negative association between duration of diabetes and AA, confirming that the length of duration of diabetes does affect accommodative functions. However, our findings are consistent with previous studies and investigations (Abokyi et al., 2016) that reported no association, potentially indicating that changes in accommodative amplitude in diabetes may be attributed to reduced lens elasticity, a consequence of the metabolic disease itself.

The present investigation found a significant decrease in both monocular and binocular AF in FHD+ individuals, with differences of approximately 1.7 and 2.8 cycles per minute (cpm) between groups. This finding aligns with Nabovati *et al*. (2022), which reported a significant association between fasting plasma glucose levels and accommodative facility. Healthy subjects with FHD+ exhibited lower accommodative amplitude and facility than expected for their age, which could potentially be serving as a new biomarker sign of the new onset of diabetes. These data, including our findings, show diabetic patients were in a short-term diabetic state. If an association exists, then we need longitudinal studies to confirm it.

Several investigators who have evaluated the accommodative functions using the push-up method in diabetes (Abokyi et al., 2016; Adnan et al., 2014; Braun et al., 1995) revealed that certain independent risk factors are accountable for reduced amplitude of accommodation. The independent risk factors included longer duration of diabetes, higher HbA1c levels, higher fasting glucose levels, and increasing severity of retinopathy. Meanwhile, Adnan *et al*. (2014) evaluated the effect of diabetes on accommodative functions and found a strong correlation between the duration of diabetes and reduced amplitude of accommodation. In that study and in the current study, the duration of diabetes was strongly associated with reduced accommodative amplitude.

Healthy subjects with a family history of diabetes who have not yet reached diabetes-threshold glucose levels showed marked reductions in accommodative functions. The observed impaired accommodative functions in the FHD+ group may be attributed solely to glucose level changes, potentially resulting from alterations in lens structure or metabolism that might be similar to those seen in diabetic individuals. A possible explanation for these changes is excess accumulation of glucose in the crystalline lens during periods of glucose dysregulation (Mathebula, 2015; Mathebula and Makunyane, 2017). The aldose reductase enzyme converts this glucose into sorbitol, which is then converted into fructose by sorbitol dehydrogenase (Mathebula, 2015). This leads to the buildup of metabolites like sorbitol and fructose within the lens, causing lenticular changes of it (Mathebula, 2015). The accumulation of sorbitol in the lens increases osmotic stress and damage to the crystalline lens (Díaz-Flores et al., 2004; Mathebula, 2015).

Another possible mechanism for accommodative anomaly involves alterations in lens morphology, as diabetics tend to have thicker and more curved lenses compared to healthy individuals. Consistent with findings by Mathebula and Makunyane (2017), our study may observe increased lens thickness and curvature in diabetic subjects, potentially contributing to reduced accommodative function. This decline in accommodative functions may be attributed to structural changes and loss of elasticity, as supported by Fisher (1988), who found diabetic lenses exhibit reduced elasticity due to alterations in the lens capsule and substances, impairing accommodative function. Sırakaya *et al*. (2020) suggest a link between fasting plasma glucose levels significantly contributing to the development of accommodative anomaly in diabetic subjects. Taking this perspective into account, it implies that the lens appears to play a significant role in the development of accommodative anomaly in DM and FHD+ subjects, and the observed decline in accommodative function might be due to accommodative anomaly rather than presbyopia, suggesting alternative factors like HbA1c may be influential and warranting further investigation into the relationship between glucose levels and accommodative function. The main contribution of this study is to note that AA is not only the parameter affected; monocular and binocular AF are also altered in both FHD+ and DM groups, compared to HC.

The study had several limitations. First, accommodative response and amplitude of accommodation were influenced by depth of focus. Small pupils increase depth of focus, which can lead to overestimating the amplitude of accommodation. Individuals with DM typically have smaller pupils than non-diabetics due to sympathetic neuropathy, which causes pupillary dilation impairment (Smith and Smith, 1983). Therefore, future research should employ objective methods for controlling pupil diameter to validate our results. Another limitation was our inability to determine the cause of accommodative dysfunctions in healthy subjects with a family history of diabetes, as we only took measurements at a single point. Furthermore, relying solely on comparing means could produce misleading results due to the potential influence of outliers and our limited sample size.

## Conclusion

The findings revealed that FHD+ who exhibited three or more pre-diabetes risk factors showed notable accommodative anomalies, although not to the same extent as those with DM. Specifically, while DM subjects had significantly worse accommodative function, the FHD+ group demonstrated a decline in accommodative amplitude and insufficiency that was intermediate between DM and healthy controls. This observation is particularly striking given that the FHD+ group showed significant differences in accommodative parameters compared to HC, irrespective of age, even with preserved visual acuity. It is anticipated that FHD+ are at a greater risk for the onset of the condition and may experience accommodative anomalies typical for their age, which could serve as a new biomarker sign of diabetes compared to individuals without a familial predisposition to diabetes. Monitoring and screening individuals with a family history of diabetes who are predispose to prediabetic stage could be beneficial, as understanding when these diabetes related-ocular changes begin or start during the progression of the disease may enhance preventive strategies in diabetes management.
